# The Orchid Velamen: A Model System for Studying Patterned Secondary Cell Wall Development?

**DOI:** 10.3390/plants10071358

**Published:** 2021-07-02

**Authors:** Nurul A. Idris, Maketelana Aleamotuʻa, David W. McCurdy, David A. Collings

**Affiliations:** 1 Faculty of Science and Marine Environment, Universiti Malaysia Terengganu, Kuala Terengganu 21030, Malaysia; nurul.aliaa@umt.edu.my; 2 School of Biological Science, University of Canterbury, Private Bag 4800, Christchurch 8140, New Zealand; 3 School of Environmental and Life Sciences, The University of Newcastle, Callaghan, NSW 2308, Australia; maketalena.akauola@uon.edu.au (M.A.); david.mccurdy@newcastle.edu.au (D.W.M.); 4 School of Molecular Sciences, The University of Western Australia, Crawley, WA 6009, Australia; 5 Harry Butler Institute, Murdoch University, Murdoch, WA 6150, Australia

**Keywords:** lignin, lignification, microtubules, orchid velamen, secondary cell wall

## Abstract

Understanding the mechanisms through which plants generate secondary cell walls is of more than academic interest: the physical properties of plant-derived materials, including timber and textiles, all depend upon secondary wall cellulose organization. Processes controlling cellulose in the secondary cell wall and their reliance on microtubules have been documented in recent decades, but this understanding is complicated, as secondary walls normally form in the plant’s interior where live cell imaging is more difficult. We investigated secondary wall formation in the orchid velamen, a multicellular epidermal layer found around orchid roots that consists of dead cells with lignified secondary cell walls. The patterns of cell wall ridges that form within the velamen vary between different orchid species, but immunolabelling demonstrated that wall deposition is controlled by microtubules. As these patterning events occur at the outer surface of the root, and as orchids are adaptable for tissue culture and genetic manipulation, we conclude that the orchid root velamen may indeed be a suitable model system for studying the organization of the plant cell wall. Notably, roots of the commonly grown orchid *Laelia anceps* appear ideally suited for developing this research.

## 1. Introduction

The primary component of the plant cell wall is cellulose, a β-1,4 polymer of D-glucose crystallized into microfibrils that contain 18 (to 24) separate glucan chains [1,2]. The organization of this cellulose within the cell wall controls plant morphogenesis: for plant cells undergoing expansion with a thin, deformable primary cell wall, the transverse orientation of the cellulose microfibrils dictates the biophysical properties of the wall and limits expansion to a direction perpendicular to the microfibrils [3]. In mature, nonexpanding plant cells, cellulose is instead organized into oblique or cross-laminate patterns that prevent further expansion. A subset of plant cells, however, develop a secondary cell wall in which extra cellulose is reinforced with the organic polymer lignin [4,5]. In these secondary walls, the organization of the cellulose again remains critical for the strength of the cell walls, and thus of the overall plant tissue [3]. These secondary cell walls are of economic importance: plant-based fibres from the finest cottons to the coarsest industrial fibres are all derived from various types of secondary cell walls and have properties dependent on cellulose organization [6]. Similarly, the properties of timber and paper made from wood pulp depend on the organization of the cellulose that makes up the walls of the xylem cells that form the wood used [7,8].

The deposition of cellulose within the plant cell wall is controlled, in part, by cortical microtubules, as demonstrated by live cell analyses that show cellulose synthase complexes which synthesize new cellulose microfibrils moving along the microtubules in expanding primary cells [9,10]. While similar studies have demonstrated a role for microtubules in secondary cell wall development [11,12], these live cell imaging experiments have been hampered by secondary cell wall development occurring in internal tissues, which has limited the clarity of the imaging conducted. Various systems have been developed that offer more tractable systems with which to study secondary cell wall formation. While classic cell culture systems, such as *Zinnia elegans* mesophyll cells that can be induced to form xylem [13], are nontransformable, *Arabidopsis thaliana* suspension cells expressing fluorescent fusions can be induced to undergo secondary cell wall development [14]. More recently, inducible systems have been developed in which the epidermal cells of *A. thaliana* roots are genetically reprogrammed to develop secondary cell walls and become xylem vessels [15,16,17,18]. While these experimental systems allow for the dissection of the roles played by microtubules in secondary cell wall formation, they remain artificial, as the cells are in culture and/or are triggered to develop through nonstandard pathways.

**Figure 1:**
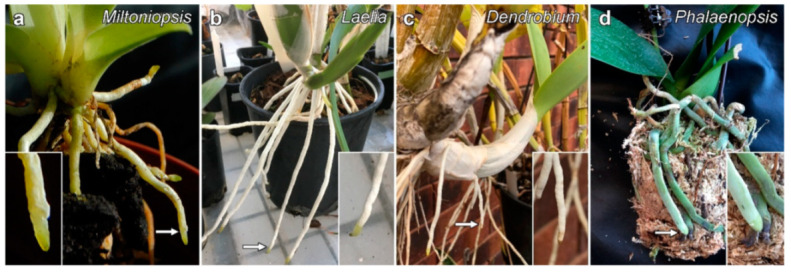
Velamen development in orchid roots. Orchid roots are covered by a thin layer of dead cells called the velamen which appears an off-white colour. Junctions between the newly formed velamen and the growing root are indicated by arrows and are enlarged in the insets. (**a**) *Miltoniopsis* sp. (**b**) *Laelia anceps*. (**c**) *Dendrobium* sp. (**d**) *Phalaenopsis* sp.

We have previously investigated microtubules and secondary cell walls in the roots of the orchid *Miltoniopsis*. These experiments demonstrate that microtubules align with developing phi thickenings in the root cortex [19], these thickenings being bands of secondary cell wall that are induced in the root cortex of numerous species including orchids [20], while preliminary observations demonstrated that microtubules are aligned with developing secondary cell wall striations in the root velamen [21]. In this study, we have expanded our observations of microtubules during velamen development, and we demonstrate that the outer cell layer within the velamen may be a useful system in which to investigate the role of the cytoskeleton in secondary cell wall formation. The velamen is a layer of dead cells with secondary wall striations that surrounds the cortex of orchid roots (Figure 1). In terrestrial orchids, the velamen typically contains only a single cell layer and coats the parenchymatic cortex. In epiphytic orchids that are prone to dehydration because their aerial roots typically only cling to the host tree’s branches surrounded by leaf litter rather than actual soil, the velamen is a specialized, spongy, multilayered structure [22,23,24,25]. This velamen is thought to function in water capture by the root [24], and in water retention in dry conditions [23,26], with the unusual nature of the velamen cell wall thought to aid in these roles. Classification of the different secondary cell patterns formed in the velamen has defined 11 different velamen patterns [24], allowing taxonomic keys to utilise velamen patterning as a defining characteristic [27,28,29]. In several of these classes, velamen wall thickenings do not show banding patterns, but some taxa show patterned wall thickenings in the velamen with helical or transverse ridges that bear superficial similarities to the wall thickenings of primary xylem.

We have previously demonstrated that the *Miltoniopsis* velamen exhibits the classic cymbidium-type velamen organization, with thin helical wall thickenings that show a webbed or mesh structure. Microtubules align along the length of these thickenings as they develop [21]. In this study, we wished to determine whether the orchid velamen might be developed as a model system in which live cell imaging can be used to investigate the role of the cytoskeleton in the formation of secondary cell walls. As a first step in this process, we surveyed cell wall organization in a range of commonly grown orchids and demonstrated that the outermost wall of the velamen shows striking patterns in several different orchids. We then investigated the role of microtubules in velamen development using conventional immunofluorescence microscopy. In all cases where microtubules were successfully imaged, they aligned along the developing cell wall ridges, but as these structures matured, the microtubules assumed a flanking position, a pattern also seen in developing vascular elements. Furthermore, as live cell imaging requires readily accessible outer epidermal and subepidermal layers, we placed extra emphasis on observing cell wall structures in the outermost cells of the velamen that would be most readily seen in such experiments. We demonstrate that in *Laelia anceps*, the outer face of the velamen has strongly patterned cell wall ridges and that strongly bundled bands of microtubules initially run parallel to these ridges before flanking the ridges on either side. Thus, the *Laelia* velamen may prove to be an interesting system in which to further study the cytoskeleton and cell wall development.

## 2. Results

Although a range of different orchid species were initially investigated, we eventually limited imaging to four different species which show different classes of velamen thickening [24]. These included *Miltoniopsis*, which we had previously investigated, and three other species, *Laelia anceps* and commercial hybrids of *Dendrobium* and *Phalaenopsis*.

### 2.1. Velamen Structure in Miltoniopsis sp.

**Figure 2:**
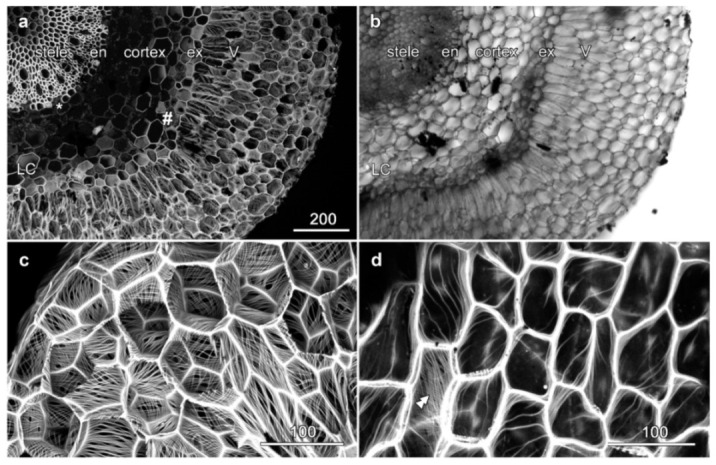
Velamen organization in *Miltoniopsis* sp. roots. Images (**a**,**c**,**d**) are maximum projections of confocal optical series showing basic fuchsin-stained lignin. (**a**,**b**) Cross section showing lignified cell walls and a concurrent transmitted light image. The wide velamen (V) surrounds the lignified exodermis (ex) and the cortex which contains lignified cortical cells (LC), while a lignified endodermis (en) surrounds the central stele. Passage cells through the endodermis (*) and velamen (#) walls are marked. (**c**) Higher resolution imaging of the criss-crossed cell wall thickenings in the velamen. (**d**) A longitudinal section shows only scattered wall thickenings in the outer wall of the outer velamen (asterisks). A region where the inner face of these cells was imaged, and which shows the criss-crossed pattern, is indicated by a double arrowhead. See also Supplementary Movies 1 and 2. Bar in (**a**) = 200 µm for (**a**,**b**); bars in (**c**,**d**) = 100 µm.

Cross sections through the *Miltoniopsis* root stained for lignin with basic fuchsin showed multiple, lignin-reinforced secondary cell walls (Figure 2). At the centre of the root, the stele was heavily lignified (Figure 2a) and surrounded by a strongly lignified endodermis (en), although passage cells (*) through the endodermis remained unlignified. The outer cortex contained some lignified cells that had little cell wall patterning (Figure 2a, LC) but no phi thickenings were present. The velamen was separated from the root cortex by the exodermis (ex) in which the hexagonally shaped cells are strongly lignified, alternating with passage cells that had a thin, mostly unlignified wall (#). However, all exodermis cells had thickened walls separating the exodermis cells and velamen cells.

The velamen surrounding the cortex showed a cymbidium-type velamen organization and consisted of six to eight layers of dead cells with larger, radially extended inner velamen cells and smaller outer velamen cells (Figure 2a,b, V). Mature velamen cell walls were reinforced by long strands of cellulose forming a mesh-like appearance (Figure 2c), although this criss-crossing effect is due to adjacent cells having cellulose patterns running in different orientations: in individual cells, the wall ridges were typically evenly spaced and roughly parallel, but their overall orientations showed no preferred direction (Figure 2c, Supplementary Movie S1 in Supplementary Materials). Longitudinal sections demonstrated that the cell wall organization in the outer velamen layer was different, and instead of the highly regular patterns of cell wall ridges present in the inner velamen, these outer cells contained only a few striations that were more irregular (Figure 2d, asterisk; Supplementary Movie S2 in Supplementary Materials).

### 2.2. Velamen Structure in Laelia anceps

**Figure 3:**
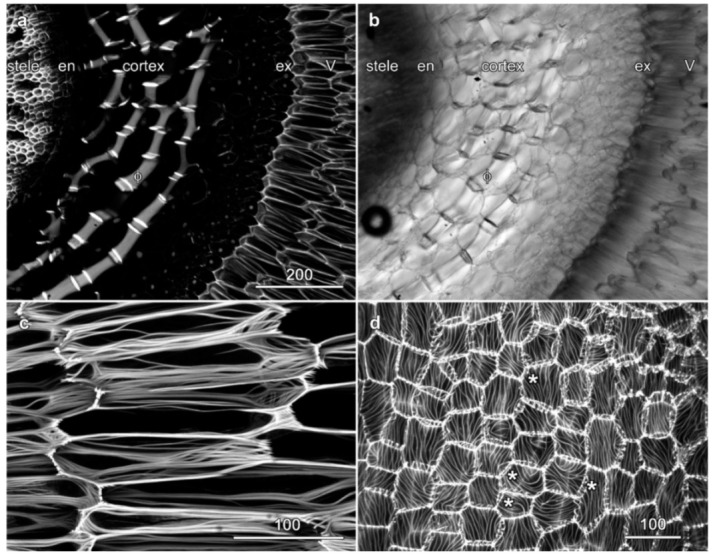
Velamen organization in *Laelia anceps* roots. Images (**a**,**c**,**d**) are maximum projections of confocal optical series of berberine hemisulfate (**a**) and basic fuchsin staining (**c**,**d**). (**a**,**b**) Cross section showing lignified cell walls, along with a concurrent transmitted light image. The velamen (V) surrounds the lignified exodermis (ex) and the cortex. Extensive phi thickenings (ϕ) are present within the cortex. (**c**) Higher resolution image of cell wall thickenings in the highly elongated inner velamen cells. (**d**) Longitudinal section showing wave-like wall thickenings in the outer surface of the velamen. See also Supplementary Movies S3 and S4. Bar in (**a**) = 200 µm for (**a**,**b**); bars in (**c**,**d**) = 100 µm.

Basic fuchsin-stained cross sections of *Laelia anceps* roots revealed numerous strongly lignified secondary walls (Figure 3). The most dramatic structures were the near-complete cortical rings of phi thickenings that encircled the stele (Figure 3a,b, Φ). While the central stele was strongly lignified, neither the endodermal cells nor the exodermal cells of *Laelia* were lignified (Figure 3a, en and ex), although the outer exodermal wall that joins to the velamen did show lignification. The organization of the velamen in *Laelia anceps* was typical of the epidendrum pattern, with three or four layers of long and narrow cells that were reinforced by thickened bands of cellulose that were broadly parallel (Figure 3c, Supplementary Movie S3 in Supplementary Materials) and with outer velamen cells that were smaller and not elongated. Longitudinal sections showed that this cell wall banding pattern continued through to the outer surface of the root where the patterning of the parallel bands was striking and showed evidence of organization between cells (Figure 3d, Supplementary Movie S4 in Supplementary Materials). Several locations where these outer velamen wall ridges ran at similar orientations in adjacent cells are highlighted in Figure 3d with asterisks.

### 2.3. Velamen Structure in Dendrobium spp.

**Figure 4:**
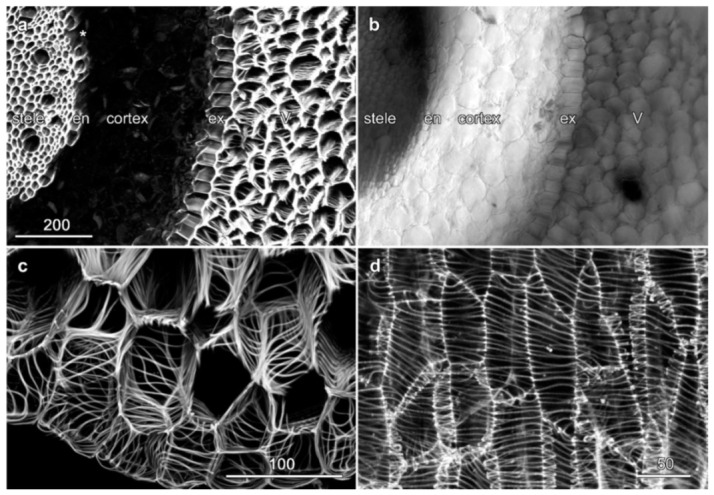
Velamen organization in *Dendrobium* sp. roots. Images (**a**,**c**,**d**) are maximum projections of confocal optical series collected from basic fuchsin fluorescence. (**a**,**b**) A slightly off-angle cross section showing lignified cell walls, along with a concurrent transmitted light image. The velamen (V) surrounds the lignified exodermis (ex) and the cortex. The partially lignified endodermis (en) with multiple passage cells (*) surrounds the central stele. (**c**) Higher resolution image of crossed cell wall thickenings in the outer velamen. (**d**) Longitudinal section showing velamen cells with transverse wall thickenings on the velamen’s outer face. See also Supplementary Movies 5 and 6. Bar in (**a**) = 200 µm for (**a**,**b**); bar in (**c**) = 100 µm.; bar in (**d**) = 50 µm.

Cell wall labelling with basic fuchsin of root sections through a *Dendrobium* hybrid showed a wide cortex, a velamen with three to five cell layers and a heavily lignified endodermis with unlignified passage cells (Figure 4a,b). The velamen showed helically arranged wall thickenings characteristic of the dendrobium class (Figure 4c, Supplementary Movie S5 in Supplementary Materials) which, in longitudinal sections, were widely spaced and oriented in a transverse pattern (Figure 4d, Supplementary Movie S6 in Supplementary Materials).

### 2.4. Velamen Structure in Phalaenopsis sp.

**Figure 5:**
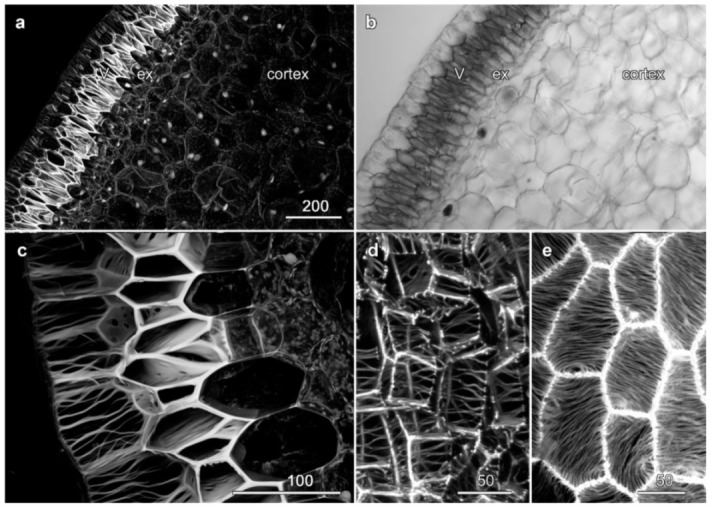
Velamen organization in *Phalaenopsis sp.* roots. Images (**a**,**c**–**e**) are maximum projections of confocal optical series collected of basic fuchsin labelling. (**a**,**b**) Cross section showing lignified velamen (V) but unlignified exodermis (ex) walls, along with a concurrent transmitted light image. (**c**) Higher resolution image of cell wall thickenings in the velamen. (**d**) Longitudinal sections through the outer face of the velamen showed either irregular cell wall ridges (**d**) or more commonly, narrowly spaced, broad swirls of secondary walls (**e**). See also Supplementary Movies S7 and S8. Bar in (**a**) = 200 µm for (**a**,**b**); bar in (**c**) = 100 µm.; bars in (**d**,**e**) = 50 µm.

The commercial *Phalaenopsis* hybrid imaged in these experiments had a velamen layer typical for the vanda class in that the velamen was only one or two cell layers thick (Figure 5a,b, Supplementary Movie S7 in Supplementary Materials). In the vanda type, velamen cells are commonly smaller than the exodermis cells that are thickened in their tangential and radial (partly thickened) walls. Longitudinal sections through the *Phalaenopsis* velamen showed varying cell wall patterns in the outer layer (Figure 5c). The outer velamen showed a considerable degree of variability: while some cells showed widely spaced bands of near transverse cellulose ridges (Figure 5d), most cells contained closely spaced, textured cell walls in which the broad thickening bands appeared to weave into and out of the wall in a wickerwork-like pattern (Figure 5e, Supplementary Movie S8 in Supplementary Materials). 

### 2.5. Cytoskeletal Organization during Velamen Development in Miltoniopsis

**Figure 6:**
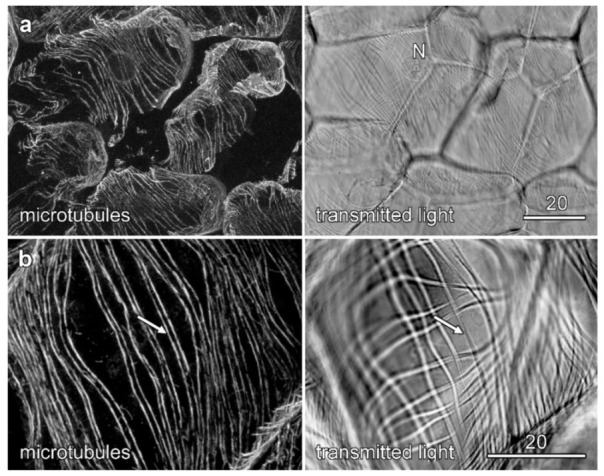
Microtubules and velamen development in *Miltoniopsis*. Microtubule images (left column) are maximum projections of confocal optical series from early (**a**) and late (**b**) velamen development and are shown alongside concurrent transmitted light images (right column). Labelling is of cells from the inner velamen in vibratome sections. When cell wall ridges first developed (**a**), they were only weakly visible by transmitted light but, being unlignified, were not yet autofluorescent. Microtubules formed strongly bundled, parallel arrays. Later in development, when cell wall ridges were more pronounced (**b**), the immunolabelled microtubules were often paired, running along either side of the cell wall ridges (arrows). Bars in (**a**,**b**) = 20 µm.

**Figure 7:**
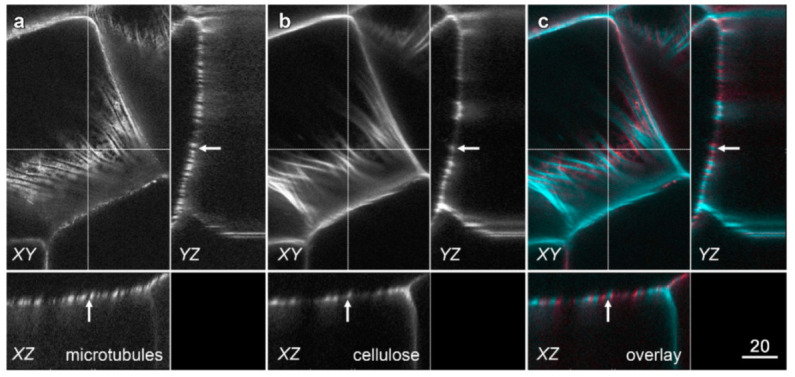
Split microtubule bands lie on either side but not underneath cell wall ridges. The microtubule splitting pattern in *Miltoniopsis* roots does not occur because of a change in focal plane of the microscope. Confocal optical sections were collected at 1 µm intervals through vibratome sections of inner velamen cells immunolabelled for (**a**) microtubules (coloured red in the overlay image) and (**b**) stained with pontamine for cellulose (coloured cyan in the overlay image). (**c**) Overlay image. Microtubules showed distinct patterns from the developing cellulose wall ridges. A single confocal optical section is shown (panels *XY*) with *YZ* and *XZ* planes being computer-generated orthogonal reconstructions at the locations indicated with the dotted lines. These reconstructions show that late in velamen development, microtubules run on either side of the secondary cell wall ridge (arrows) rather than directly underneath them. Bar in (**c**) = 20 µm for all images.

Microtubule immunolabelling was conducted on formaldehyde-fixed tissue from near the tip of the root where velamen development was occurring (Figure 1). The two methods used to allow antibody penetration through the cell wall were sectioning with a vibratome and enzymatic digestion of root sections. While sectioning generally gave more consistent labelling patterns, enzymatic digestion of the walls proved superior when viewing the outer cortical cells and, in particular, the outer face of the velamen. As previously documented [21], tubulin immunolabelling of Vibratome sections of *Miltoniopsis* roots showed that microtubules were strongly bundled early in the development of the velamen when the cell wall ridges were only just visible with transmitted light (Figure 6a). Later in velamen development, as shown by the strong development of wall ridges in the transmitted light image, bundled microtubules lay parallel to the cell wall ridges, with paired microtubule bundles flanking either side of the wall ridges (Figure 6b). Orthogonal sections generated in ImageJ through optical stacks showed that these paired microtubule bundles ran adjacent to the wall ridges and did not lie beneath them (Figure 7, arrows). 

### 2.6. Microtubules and Velamen Development in Laelia 

**Figure 8:**
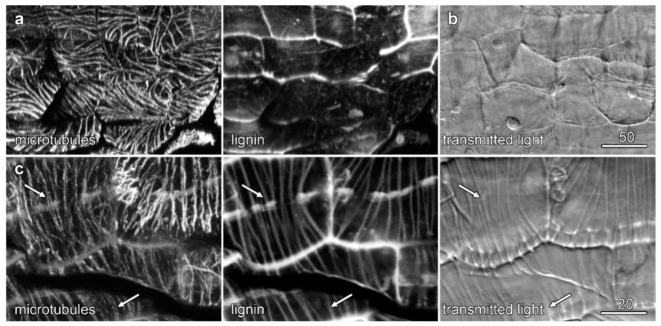
Microtubules and velamen development in *Laelia*. Labelling of the outermost layer of the velamen was achieved using cell wall digestion enzymes. Confocal maximum projections of microtubules (left-hand column) and lignin autofluorescence (central column), along with concurrent transmitted light images (right-hand column). (**a**,**b**) Early in development, microtubules have become bundled, and while cell wall ridges were visible in transmitted light, they had not become lignified. Microtubules ran parallel to the wall ridges as single bundles. Confocal images of lignin and microtubules (**a**) were reoriented so that the outer cell layers lie in the image plane, while the transmitted light image (**b**) remains unrotated. (**b**) Once cell wall ridges had developed lignification, the microtubule bundles split to run on either flank of the ridges (arrows). Bar in (**a**) = 50 µm for (**a**,**b**); bar in (**c**) = 20 µm.

Immunolabelling experiments in the developing *Laelia* root used enzyme-digested sections to visualise microtubules in the outermost cells of the velamen, where the most dramatic cell wall organization had been observed (Figure 3d) and showed similar patterns of microtubule organization to those seen in *Miltoniopsis*. In younger cells, nearer the root tip, microtubule bundling preceded the lignification of the cell wall ridges (Figure 8a), whereas in older cells, between 1 and 2 mm further away from the root tip, lignification of the ridges had begun, and the microtubule bundles which had been whole showed evidence of splitting so that microtubules flanked the cell wall ridges (Figure 8c, arrows). We have attempted microtubule immunolabelling in *Dendrobium* roots, as these also showed striking patterns in the outermost velamen wall, but to date, labelling experiments have not proven to be successful. 

### 2.7. Microtubules and Velamen Development in Phalaenopsis

**Figure 9:**
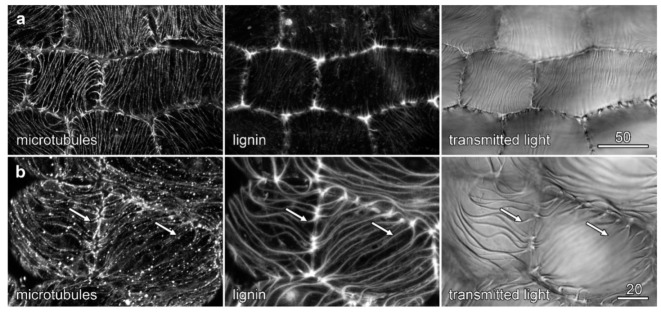
Microtubules and velamen development in *Phalaenopsis*. Labelling of the outermost layer of the velamen was achieved using cell wall digestion enzymes. Confocal maximum projections of microtubules (left-hand column) and lignin autofluorescence (central column), along with concurrent transmitted light images (right-hand column). (**a**) Early in development, microtubules have become bundled, and while cell wall ridges were visible in transmitted light, they had not become lignified. Microtubules ran parallel to the wall ridges as single bundles. (**b**) Once cell wall ridges had developed lignification, the microtubule bundles split to run on either side of the ridges (arrows). Bar in (**a**) = 50 µm; bar in (**b**) = 20 µm.

Cell wall digestion of root sections was also used to observe the outer face of the velamen. While strongly bundled microtubules were seen early in velamen development, again prior to the development of significant cell wall lignification (Figure 9a), observing microtubules in older parts of the root proved to be problematic, presumably due to suboptimal enzyme digestion. However, in cases where labelling was observed, the microtubule flanking patterns were sometimes observed (Figure 9b, arrows). Imaging further highlighted the variability in the outer wall of the *Phalaenopsis* velamen (compare lignin patterns in Figure 9 to Figure 5).

## 3. Discussion

The velamen is a specialized tissue in the outer layer of the orchid root, having cell walls with patterned, secondary cell wall thickenings. This cell layer, which is most prominent in epiphytic orchids, is an adaptation that optimizes water retention [23,26] and water and nutrient intake from the environment [25], both of which are important factors for epiphytic orchids whose roots are not typically under moist soil. Although such wall thickenings are almost unique to orchid roots, similar patterned cell walls have also been observed covering the roots of several other families of plants, including the fern *Asplenium* [30] and related species [31]; the Cyperaceae and Velloziaceae, where thickenings are multilayered [24]; and other monocots [32].

### 3.1. Microtubules and Velamen Development

The secondary thickenings of the orchid velamen cell wall consist of cellulose microfibrils orientated in specific patterns, with the organization of these patterns being an important characteristic in taxonomic identifications [27,33]. Our observations of four different orchid species showed patterns consistent with these previous characterizations. The velamen develops from living postmeristematic cells that undergo secondary cell wall deposition and subsequent programmed cell death to leave the shell of the cell wall. The coalignment of cellulose deposition and microtubules is well known [3], and cellulose synthase complexes that synthesize cellulose microfibrils coalign with and travel along microtubules in both primary [9,33] and secondary cell walls [11,12], so it is no surprise that microtubules ran parallel to the deposition of the cellulose ridges during velamen development in orchid roots.

A single, previous study of velamen cell wall development in *Ansellia* used transmission electron microscopy to report the association of microtubules with the later stages of wall thickening development, but no evidence was observed for “cytoplasmic pre-patterning” or alignment of microtubules with the early stages of wall thickening development [33]. However, our observations, reported in a preliminary form in *Miltoniopsis* [21] and repeated more extensively in this study for a wider range of species, have confirmed extensive microtubule alignment with wall thickenings, with apparently strongly bundled microtubules aligning to the ridges formed due to the secondary deposition of cellulose microfibrils. Thus, cytoskeletal organization in velamen cells is consistent with other secondary cell wall thickenings where microtubules coalign with wall deposition, including tracheary elements in various plants [12,13,14,34,35,36] and cortical phi thickenings [19].

### 3.2. The Unusual Pattern of Microtubules That Flank the Cell Wall Ridges

The observation that microtubules initially align with the developing secondary wall ridges and then develop into a pattern where parallel microtubules flank the ridges as the velamen becomes more mature is intriguing. We observed this pattern previously in *Miltoniopsis* [21], and it was observed in all four orchid species used in this study. Moreover, orthogonal sectioning demonstrated that these microtubules truly flanked the cell wall ridges and were not a single band of microtubules that was partially out of the focal plane. This flanking microtubule pattern might best be explained by microtubules being involved in widening the cell wall ridges once they have formed. This flanking pattern has been reported before during secondary wall development of tracheary cells by immunofluorescence in *Zinnia* tissue culture cells that develop into vessel elements [13,34], with GFP-based probes in *Arabidopsis* tissue cultures also differentiating into xylem [14] and during xylem differentiation in *Arabidopsis* roots [12]. Similar flanking microtubules have also been observed around the cell wall ridges (ingrowths) deposited during the development of xylem transfer cells in wheat [37]. Thus, the secondary wall formation of velamen cells is similar to that of the xylem and other secondary cell walls, with aligned microtubules directly overlaying secondary wall deposition during the initial stages of secondary thickening formation and subsequently reorganizing to flanking positions at later stages.

### 3.3. Developing Orchid Roots as a Model System for Secondary Cell Wall Investigations?

Orchid roots, and the velamen in particular, provide an interesting system in which to study various developmental paths. Recently, for example, velamen development was used to investigate the control of programmed cell death [38], while the velamen has also been suggested as a model system for investigating the uptake and flow of water through porous material [26]. In this study, we used immunofluorescence microscopy to answer the question as to whether velamen formation in orchid roots might be developed as a model system for live cell imaging of secondary cell wall formation. Such a model system might, ideally, have the following characteristics:

(i) secondary cell wall development occurring in a developmentally recognizable sequence at specific sites;
(ii) secondary cell wall development showing similar characteristics to ‘important’ secondary cell walls, such as xylem development;
(iii) secondary cell wall development in surface layers that are readily accessible for microscopy;
(iv) a system readily adapted to tissue culture;
(v) the ability to stably transform tissues so that they express fluorescent protein constructs;
(vi) the availability of genomic information and access to mutants;
(vii) and limited autofluorescence that would confound live cell imaging experiments.

No currently used experimental system displays all these characteristics, with lignin autofluorescence associated with secondary wall development being problematic across a broad range of wavelengths [39], although the recent development of transgenic *Arabidopsis* lines in which epidermal cells can be reprogrammed to develop secondary cell walls with the use of inducible promoter systems [16,17,18] comes close. However, the orchid velamen system, and in particular *Laelia anceps*, would provide some advantages as a model system for secondary cell wall studies, especially if longer wavelength fluorescent proteins, such as mCherry or the recently developed near infrared proteins [40], were used. Not only are plants commercially available and readily grown in tissue culture, but the velamen also has highly visible secondary thickenings in a cell layer readily accessible at the root surface. The development of these cell wall ridges is controlled by the organization of the microtubules within the cells and follows patterns consistent with xylem development, and as with developing tracheary cells, the velamen cells then undergo programmed cell death. 

While the experiments conducted to date have used immunofluorescent labelling of fixed tissues to investigate microtubule development and were thus limited in scope because of the need to either use sectioned material or cell wall digests—as was the case for observing the outer layers of the velamen—the best understanding of the roles for microtubules in cell wall formation would require live cell imaging. Fusions of fluorescent proteins to tubulin and cellulose synthase have redefined our understanding of cell wall formation in both primary and secondary cell walls [9,11,12]. For the orchid root system to be useful for such live cell imaging experiments, transformation would be required. Fortunately, extensive protocols for growing and splitting orchid plants in culture exist, and a range of orchid species has now been stably transformed using a combination of gene gun- and *Agrobacterium*-based approaches, including *Cymbidium* [41], *Dendrobium* [42], *Oncidium* [43] and *Phalaenopsis* [44]. Additionally, while neither of the orchid species (*Phalaenopsis equestris* and *Dendrobium catenatum*) for which genomes are publicly available [45,46] proved suitable for imaging when tested, several further genomes have been published more recently (*Dendrobium officinale* and *Apostaceae shengen*) [47], with the likelihood of more being published in the near future.

## 4. Materials and Methods 

### 4.1. Plant Material

Various orchid species (Table 1) were purchased from commercial growers and local suppliers and grown in orchid mix (Osmocote, Scotts Australia, Bella Vista NSW, Australia) in a greenhouse at 24 °C/18 °C (day/night) under partial shade.

### 4.2. Fixing Root Tissue

Roots were fixed in PME solution (50 mM Pipes pH 7.2, 2 mM EGTA, 2 mM MgSO_4_) supplemented by 0.1% (*v/v*) Triton X-100, 3.7% (*v/v*) formaldehyde and 0.1% (*v/v*) dimethyl sulfoxide for a minimum of 1–2 h [48]. Excised roots were vacuum infiltrated with fixative for at least the first 15 min to aid penetration of the fixative through the velamen and into the root. Prior to analysis, fixed roots were washed in phosphate buffered saline (PBS; 131 mM NaCl, 5.1 mM Na_2_HPO_4_, 1.56 mM KH_2_PO_4_, pH 7.2).

### 4.3. Cell Wall Labelling

Hand sections of fixed root material were rinsed in distilled water and stained for lignin with either berberine hemisulfate (0.1% (*w/v*) in water, 5 min, Sigma, St. Louis, MO, USA) [49] or basic fuchsin (0.001% (*w/v*) in water, 5 min; Sigma) [50]. Samples were also labelled for cellulose with pontamine fast scarlet 4B (0.1% (*w/v*) in 150 mM NaCl, 5 min; Sigma) [51,52,53]. Sections were mounted in glycerol on slides.

### 4.4. Immunolabelling of the Cytoskeleton

Immunolabelling of root sections followed published procedures, with two different approaches used to allow antibodies access to fixed cytoplasm [19,21,50,54] in regions of the root tip near where the velamen was developing (Figure 1). For immunolabelling, whole roots were extracted in PME supplemented with 1% (*v/v*) Triton X-100 (1 h), permeabilized in methanol (−20 °C, 15 min) and rehydrated in phosphate-buffered saline. In the first approach, longitudinal sections about 1 mm thick were cut from the surface of roots by hand with a double-sided razor blade, and the sections treated for ~15 min with cell wall digest enzymes (1% (*w/v*) pectoylase YC, 0.2% (*w/v*) macerozyme, 1.0% (*w/v*) BSA and 0.1% (*v*/*v*) Tween-20 dissolved in PBS buffered to pH 6.0). This approach allowed for antibody labelling of the cells in the outermost layer of the velamen. Alternatively, ~100 µm thick sections were cut after whole roots were embedded in polymerized acrylamide gel (21% (*v/v*), Biorad, Hercules, CA USA) [19], an approach that was suitable for labelling cells in the inner part of the velamen. After washing in PBS, sections were blocked in incubation buffer (PBS containing 1% (*w/v*) bovine serum albumin (BSA) and 0.5% (*v/v*) Tween-20, 15 min) before being incubated in mouse monoclonal anti-α-tubulin (clone B512, Sigma, diluted 1/1000 in incubation buffer, 1 h). After multiple washes in PBS, sections were incubated in a mixture of secondary antibodies (goat antimouse coupled to fluorescein and goat antimouse coupled to Cy5; Jackson ImmunoResearch, West Grove, PA, USA, diluted 1/200 in incubation buffer, 1 h). The use of the two secondary antibodies allowed for visual screening for microtubule labelling, looking for fluorescein fluorescence, but allowed for confocal imaging using Cy5 at a wavelength where background autofluorescence was minimized. After several washes in PBS, sections were mounted in antifade agent (type AF1, Citiflour, London, UK).

### 4.5. Confocal Microscopy

Root sections were observed by confocal microscopy with an Olympus FV1000 microscope using 10× NA 0.30 dry, 20× NA 0.60 dry and 30× NA 1.05 silicone oil immersion lenses. Fluorescence from fluorescein and berberine resulting from excitation at 473 nm was collected from 500–550 nm and basic fuchsin and pontamine were excited at 559 nm with emission collected from 570 to 620 nm, while Cy5 excited at 635 nm was imaged from 650–760 nm. Lignin autofluorescence was excited at 405 nm and imaged from 420 to 460 nm. Transmitted light images were collected concurrently in either bright-field, DIC or polarization modes. Z-stacks through multiple cell layers were collected with step sizes ranging from 0.5 to 2.0 µm and, for berberine and basic fuchsin imaging of lignin fluorescence, were collected with the pinhole minimized to improve resolution in the Z-direction. All images were processed through ImageJ (FIJI installation version 1.51j, NIH, Bethesda, MD, USA) to select appropriate cell layers and to generate three dimensional rotations, and with standard tools in Adobe Photoshop version CS4 (version 11.0.1, Adobe Systems, San Jose, CA, USA). For some experiments, a Leica SP5 confocal microscope was used with 20× NA 0.7 and 63× NA 1.3 glycerol immersion lenses. Excitation wavelengths were 488, 561 and 633 nm, and similar emission windows were used.

## 5. Conclusions

We have shown that the velamina of four different orchid species that are readily available show different patterns of secondary cell wall development and that in the case of *Laelia* and *Dendrobium*, these secondary cell walls are highly structured in the cell layer immediately at the surface of the root. The formation of these secondary cell walls is controlled by microtubules, and as with other secondary cell walls, the microtubules initially align parallel to the cell wall ridges but subsequently develop a flanking pattern running along either side of the maturing wall ridge. In conclusion, we suggest that our data demonstrate that the orchid velamen, and in particular the velamen layers of *Laelia anceps* and *Dendrobium*, may prove to be worth developing as live cell imaging systems for studying the formation of the secondary cell wall. We note, however, that the orchid velamen shows extensive patterning beyond the structures seen in this study, with 11 different velamen patterns having been defined [24]. Further surveys of orchid taxa may identify a species where not only are transformation protocols and sequence information available, but novel secondary cell walls are also present whose study might be informative concerning secondary cell walls in general.

## Figures and Tables

**Table 1:** Orchid species investigated.

Species	Velamen Thickening Class (as Defined by Porembski and Barthlott 1988) [24]
*Dendrobium* sp.	dendrobium
*Laelia anceps*	epidendrum
*Miltoniopsis* sp.	cymbidium
*Phalaenopsis* sp.	vanda

## Data Availability

The data presented in this study are available on request from the corresponding author.

## Supplementary Materials

The following are available online at https://www.mdpi.com/article/10.3390/plants10071358/s1. Supplementary Movie S1.avi: *Miltoniopsis*: animation of confocal imaging of lignin in a cross section through the velamen. Supplementary Movie S2.avi: *Miltoniopsis*: animation of confocal imaging of lignin in longitudinal section through the outer velamen. Supplementary Movie S3.avi: *Laelia*: animation of confocal imaging of lignin in a cross section through the velamen. Supplementary Movie S4.avi: *Laelia*: animation of confocal imaging of lignin in longitudinal section through the outer velamen. Supplementary Movie S5.avi: *Dendrobium*: animation of confocal imaging of lignin in a cross section through the velamen. Supplementary Movie S6.avi: *Dendrobium*: animation of confocal imaging of lignin in longitudinal section through the outer velamen. Supplementary Movie S7.avi: *Phalaenopsis*: animation of confocal imaging of lignin in a cross section through the velamen. Supplementary Movie S8.avi: *Phalaenopsis*: animation of confocal imaging of lignin in longitudinal section through the outer velamen.
